# Functional Neurologic Disorder Mimicking Acute Stroke: The Diagnostic Value of Serial Bedside Examination Despite Normal Neuroimaging

**DOI:** 10.51894/001c.165966

**Published:** 2026-07-31

**Authors:** Ebtekar Mounzer, Malitha Hettiarachchi

**Affiliations:** 1 College of Osteopathic Medicine Michigan State University

## 106

### Introduction

Acute aphasia and depressed level of consciousness are high-risk presentations requiring emergent evaluation for ischemic stroke and other time-sensitive neurologic conditions. However, stroke mimics account for a substantial proportion of stroke activations, with functional neurologic disorder (FND) representing an important etiology. Diagnosis is strengthened when clinicians identify positive functional signs, such as internal inconsistency, distractibility, and incongruence with neuroanatomic patterns, rather than relying solely on negative imaging. We present a stroke activation ultimately attributed to stress-related FND to highlight the diagnostic value of serial bedside reassessment.

### Case Description

A 62-year-old Spanish-speaking–only woman with hypertension and prior cerebrovascular accidents without baseline deficits presented via EMS for acute unresponsiveness and reported aphasia after last known well at 23:00. History and examination were initially limited by language barrier and decreased level of consciousness, requiring interpreter assistance and collateral information from family. On emergency department arrival, she required repeated stimulation to arouse and was assigned a National Institutes of Health Stroke Scale score of 13, prompting stroke activation; she was outside the thrombolysis window. Non-contrast CT head, CT angiography of the head and neck, and CT perfusion demonstrated no hemorrhage, large-vessel occlusion, or perfusion abnormality. Initial laboratory evaluation for metabolic, infectious, and toxic etiologies was unrevealing. She regained meaningful alertness within approximately one hour of arrival with rapid improvement in language. MRI brain showed no acute infarction, and transthoracic echocardiogram revealed no cardioembolic source. Serial neurologic examinations during hospitalization demonstrated fluctuating, nonlocalizing findings including give-way weakness, variable sensory loss not conforming to vascular territories, preserved motor function with distraction or task reframing, and transient gait impairment with complete resolution. Symptom onset followed acute psychosocial stress and chronic insomnia; psychiatry evaluated recent suicidal ideation related to bereavement without active intent.

### Discussion

This case underscores that normal neuroimaging does not conclude diagnostic reasoning during stroke activations. Serial bedside examinations revealed positive functional signs supporting FND after exclusion of structural pathology. Recognition of FND within acute stroke pathways may reduce unnecessary escalation of care while maintaining patient safety and enabling timely, validating communication and appropriate outpatient follow-up.

